# Population Genetic Divergence among Worldwide Gene Pools of the Mediterranean Mussel *Mytilus galloprovincialis*

**DOI:** 10.3390/ani13243754

**Published:** 2023-12-05

**Authors:** Yassine Ouagajjou, Adil Aghzar, Pablo Presa

**Affiliations:** 1 Amsa Shellfish Research Station, National Institute of Fisheries Research, Tetouan 93000, Morocco; ouagajjou@inrh.ma; 2 Research Team of Agriculture and Aquaculture Engineering (G2A), Polydisciplinary Faculty of Larache, Abdelmalek Essaadi University, Tetouan 93000, Morocco; a.aghzar@uae.ac.ma; 3 Laboratory of Marine Genetic Resources (ReXenMar), CIM—Universidade de Vigo, 36310 Vigo, Spain

**Keywords:** gene pools, genetic diversity, microsatellites, *Mytilus*, world distribution

## Abstract

**Simple Summary:**

The smooth-shelled marine mussel *Mytilus galloprovincialis* maintains its genetic integrity as a species on a worldwide scale. Current population genetic analyses confirm the largest divergence of *M. trossulus* compared to the rest of congeneric species and place *M. chilensis* as an intermediate taxon between *M. galloprovincialis* and *M. edulis*. Unlike previous reports, *M. galloprovincialis* from the Atlantic Northeast is the most likely source of exotic settlements worldwide. As a super-adaptive species, *M. galloprovincialis* should not be considered invasive in a human-like supremacist manner, but rather as a flexible evolutionary species (FES). The worldwide distribution of this species suggests that it is naturally endowed with plastic adaptation. Therefore, it could counteract stressful conditions and provide intergeneric ecological opportunities in the face of climatic rarefaction of world coasts.

**Abstract:**

The Mediterranean mussel *Mytilus galloprovincialis* is distributed in both hemispheres either natively or introduced. The updated population genetic distribution of this species provides a useful knowledge against which future distribution shifts could be assessed. This study, performed with seven microsatellite markers and three reference species (*M. edulis*, *M. chilensis* and *M. trossulus*), aimed to determine the scenario of genetic divergence between 15 samples of *M. galloprovincialis* from 10 localities in Europe, Africa, Asia, Australia, North America and South America. In agreement with previous data, *M. trossulus* was the most divergent taxon of the genus, but *M. chilensis* appeared as an intermediate taxon between *M. edulis* and *M. galloprovincialis*, though closer to this latter. *M. galloprovincialis* from the Atlantic Northeast appears as the most likely source of worldwide exotic settlements instead of the previously thought Mediterranean population. The successful worldwide establishment of *M. galloprovincialis* suggests it is a flexible evolutionary species (FES), i.e., a species or population whose genetic background allows it to rapidly adapt to changing environments. This natural endowed plastic adaptation makes it a candidate resilient species amidst the ongoing climatic change.

## 1. Introduction

Smooth-shelled mussels of the genus *Mytilus* are among the most cosmopolitan genera inhabiting marine and estuarine coastal areas over temperate and sub-polar regions [1]. Three *Mytilus* species from the Northern Hemisphere have been profusely studied, i.e., *M. edulis* Linnaeus 1758, *M. trossulus* Gould 1850 and *M. galloprovincialis* Lamarck 1819 [2,3]. Those species show distinct latitudinal ranges with patching patterns and hybridization at overlapping areas [4]. The temperate *M. galloprovincialis* evolved in the Mediterranean and later expanded along Atlantic Northeast shores as far as the British Isles and Northern Africa [5]. Exotically distributed populations of this species are believed to be introduced to Australia [6], Argentina [7], Brazil [8], California [9], Central Chile [10], Southeast Asia [11] and South Africa [12]. At those exotic regions, *M. galloprovincialis* hybridize with native taxa, e.g., in Australia, South America and California, i.e., with southern Hemisphere *M. galloprovincialis*, *M. edulis platensis* and *M. trossulus*, respectively.

Its widespread distribution combined with local environmental pressure on shell shape have traditionally produced a confused taxonomy within this genus [13]. Despite their interspecific hybridization and their potential planktotrophic larvae dispersal, *Mytilus* species maintain their genetic integrity over large geographical areas [14,15]. The species status was allowed to develop specific molecular tools related to commercial interests of traceability in fisheries and aquaculture [16,17,18]. Also, the reliable identification of species is a prerequisite to determine the natural or Introduced distribution of the *Mytilus* species, to detect hybridization and introgression, as well as to gauge adaptive responses of conservation pertinence [19,20]. Recent advancement of genetic technologies has provided a wide variety of specific molecular markers useful to clarify taxonomic uncertainties within *Mytilus* spp., e.g., in many parts of Australia and New Zealand [19], East Asia [21], Northern Africa [22] and South America [23]. Those studies indicate that the main genetic divergence is between species, but a great deal also exists intraspecifically [24].

In the case of *M. galloprovincialis*, global concerns about sustainable fisheries and aquaculture management have raised interest in its distribution and dispersal patterns through its native range. Such distribution is generally the byproduct of natural population dynamics for many marine shellfish, i.e., the larval dispersal ability along the coasts determines the gene flow intensity that finally shapes the metapopulation gene pool scenario [25]. For instance, the regional genetic distribution of this species has been described on the coasts of the Iberian Peninsula [26], along the crossroads between Southern Europe and Northern Africa [22,27,28], along the Mediterranean and the Black Sea [29,30] and at a local regional scale, such as Galicia, where it has a pivotal ecosystem role [31]. Phylogeographic studies have shown that regional gene pools are hardly ever genetically homogenous in their range [32]. Although selective forces prompting local adaptation cannot be excluded as causative of the regional divergence, they have rarely been experimentally shown [33]. Meanwhile, the gene flow–gene drift balance usually explains satisfactorily the metapopulation patterns observed, which generally fit isolation-by-distance scenarios, excepting those at transitional barriers between biogeographic regions [22].

Understandably, the threat that the expansion of *M. galloprovincialis* represents to local genetic resources has fueled studies on population dynamics and conservation solutions in many regions, such as South Africa [34], California [35], Brazil [36] and Chile [37]. From our personal biological perspective, this species has been too frequently demonized as one of the worst invasive species because of its rapid adaptive success to exotic locations [38,39]. Would these negative arguments still be levelled against *M. galloprovincialis* if within a few decades it became the only intertidal mussel resilient to climate change? The updated global population genetic distribution of *M. galloprovincialis* provides useful knowledge against which future distribution shifts could be assessed. To date, few studies have been conducted comprehensively on its whole range. One of them is an in silico data mining study on the mtDNA COI sequence distribution, which showed a complex dispersal pattern likely involving a combination of natural and anthropogenic dispersal, coupled with local adaptation and hybridization events [40]. A second global study dealt with the genetic background of this species for temperature resilience. Therein, authors reported that the adaptive genetic composition was significantly different among populations and is associated with temperature variables in the Northern Hemisphere [41].

If present knowledge on the distribution of *M. galloprovincialis* allows the assessment of future distribution shifts, in this study we aimed to determine the scenario of genetic divergence between 15 populations of *M. galloprovincialis* sampled from 10 localities in Europe, Africa, Asia, Australia, North America and South America. Given the conservation of nDNA microsatellite flanking regions between close congeneric species, [42] as occurs in *Mytilus* [43,44], we hypothesize the feasibility of identifying sister species, congeneric hybrids and the genetic purity of *M. galloprovincialis* groups from each geolocation, as well as the putative original sources of its actual exotic distribution.

## 2. Materials and Methods

### 2.1. Sampling

**Figure 1:**
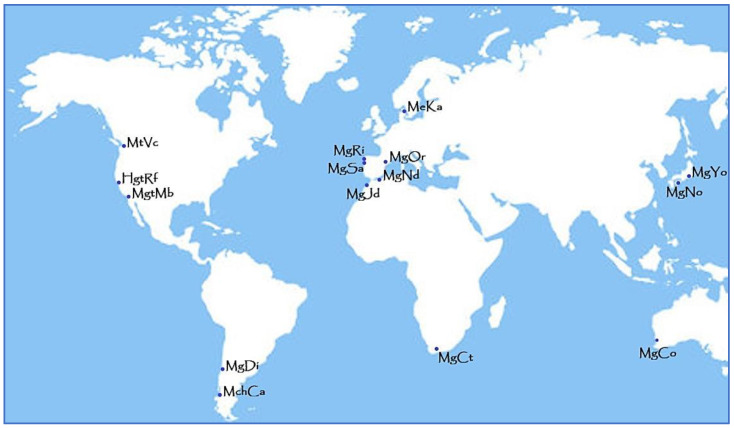
Coastal sampling locations of the *Mytilus* populations analyzed in this study. Mg, *M. galloprovincialis*; Mch, *M. chilensis*; Me, *M. edulis*; Mt, *M. trossulus* (see details in Table 1).

Aiming to screen the global genetic diversity of *M. galloprovincialis*, the sampling effort was accomplished in 2007 on intertidal areas of Southwestern Europe (Spain), North Africa (Morocco), South Africa (Cape Town), Pacific Northwest (Japan), Pacific Southwest (Australia), Pacific Northeast (California) and the Pacific Southeast (Chile) (Figure 1).

Three external reference samples of congeneric species were also included in the analyses, i.e., *M. edulis* (Denmark), *M. trossulus* (Canada) and *M. Chilensis* (Chile) (Table 1). The mantle tissue of 492 specimens were conserved in 95% ethanol until DNA extraction and purification following the FENOSALT protocol [45].

### 2.2. Molecular Analyses

All mussels were genotyped with seven polymorphic microsatellites, five of which were previously described ([43]; *Mg*µ1, *Mg*µ2, *Mg*µ3, *Mg*µ4, *Mg*µ5) and employed to genotype *M. galloprovincialis* from the Iberian Peninsula [26]. Two additional markers were employed, microsatellite *Mech*8 [44] and an unpublished one from *M. galloprovincialis*, *Mg*µ8 (forward primer 5′–ATGTCTCCTCAATCTGG–3′ and reverse primer 5′–AAATCGTTAAAAAGCAAT–3′), annealed at 55 °C and 1.7 mM MgCl_2_. PCR amplification consisted of an initial denaturing step at 95 °C for 5 min, followed by 35 cycles at 94 °C for 1 min, 1 min at the annealing temperature, and 1 min at 72 °C for extension. A final extension step was performed at 72 °C for 15 min. The amplified fragments were electrophoresed in an ALFexpress-II automatic fragment analyzer (GE Healthcare, Barrington, IL, USA) and the allele calling was helped by molecular ladders.

### 2.3. Data Analyses

The number of alleles and their frequencies, the allelic richness per locus (*R*_S_) and the fixation indexes *F* [46] were calculated with FSTAT 3.9.5 [47]. The probability test associated to *F*_IS_ was calculated with the Markov chain method implemented in GENEPOP 4.2 [48], using 20 batches of 5000 iterations each. The expected heterozygosity (*H*_E_), the observed heterozygosity (*H*_O_) and the Hardy–Weinberg equilibrium per sample were also calculated with GENEPOP. Correction for multiple tests was performed with the false discovery rate approach (FDR) [49]. Putative frequencies of null alleles co-segregating in the allelic systems and their confidence intervals were checked per locus and sample using the EM algorithm [50] and 1000 permutations, as implemented in FreeNA [51].

Fisher’s exact test and Pearson’s chi-square test were used to estimate the statistical power of the sampling system at refuting the hypothesis of genetic homogeneity (combining *t* generations of drift and effective size values to test for a specific *F*_ST_, through one batch of 1000 replicates), as well as to estimate the proportion of false significant tests (Type I error, *p* < 0.05) in combined test statistics (1000 replicates with the same effective size) as implemented in POWSIM 4 [52]. The interpopulation fixation index *F*_ST_ was calculated with FSTAT, and the differentiation parameter *D*_EST_ [53] between samples was calculated in DEMEtics 0.8–7 [54], as implemented in R–package 2.12.1., using 1000 bootstrap replicates to estimate statistical significance.

Sample relationships upon variance components on the genotype matrix were visualized in a bi-dimensional space using a principal coordinates analysis (pCoA) as available from the statistical package GenAlEx 6.5 [55]. A locus-by-locus AMOVA, as implemented in ARLEQUIN 3.5 [56], was used to distribute hierarchically the genetic variance as per six major regions (Atlantic Southwestern Europe and North Africa, South Africa, Pacific Northwest, Pacific Southwest, Pacific Northeast and Pacific Southeast) and per hemisphere. Variance distribution was also computed for congeneric species as reference samples. Statistical tests for each fixation index were based on 1023 permutations. The Bayesian inference on the number of gene pools was explored with BAPS 6 [57], considering both the allele frequencies and the number of genetically divergent groups as random variables, and either an admixture analysis based on 100,000 Bayesian iterations or a mixture model [58].

## 3. Results

### 3.1. Genetic Diversity

All seven microsatellites were polymorphic in *M. galloprovincialis* and *M. chilensis*, 86% in *M. edulis* and 71% in *M. trossulus* (Table S1). The number of alleles per locus (*A*) and the allele richness (*R*_s_) differed significantly (3000 permutation tests, *p* (two-sided) = 0.00033) between species, i.e., *M. galloprovincialis* (*Ā ± SD* = 9.986 ± 4.366; *R*_s_ = 6.857), *M. chilensis* (*Ā ± SD* = 13.429 ± 10.277; *R_s_* = 8.486), *M. edulis* (*Ā ± SD* = 7.000 ± 4.435; *R*_s_ = 6.013) and *M. trossulus* (*Ā ± SD* = 5.286 ± 3.592; *R*_s_ = 4.076) (Table S2). Thirteen species-specific alleles were observed in *M. galloprovincialis*, nineteen in *M. chilensis* and one in each of *M. edulis* and *M. trossulus*. Most of the microsatellites exhibited a heterozygosity higher than 70% over all samples, with average *H_E_* ± SD = 0.723 ± 0.183 in *M. galloprovincialis*. The values of *F*_IS_ ± SD ranged from 0.193 ± 0.116 to 0.398 ± 0.213, and genotypic disequilibrium was observed in most loci, with the putative null allele frequency averaging 0.108 ± 0.082 across samples (Table S2).

### 3.2. Genetic Differentiation

Pairwise *F*_ST_ values averaged 0.102 ± 0.044 between samples of *M. galloprovincialis* and ranged between 0.003 (MgYo–MgNo) and 0.186 (MgYo–MgOr). This distance was maximal between *M. galloprovincialis*–*M. trossulus* (0.320 ± 0.040), followed by *M. galloprovincialis*–*M. edulis* (0.165 ± 0.058) and *M. galloprovincialis*–*M. chilensis* (0.116 ± 0.043) (Table S3). All the pairwise comparisons were significant except between the two *M. galloprovincialis*–*M. trossulus* hybrid samples (MgtRf–MgtMb; *F*_ST_ = 0.003; *CI* [−0.008, 0.014]), between samples of *M. galloprovincialis* from Japan (MgYo–MgNo; *F*_ST_ = 0.003; *CI* [-0.006, 0.015]), from Spain and South Africa (MgRi–MgCt; *F*_ST_ = 0.040; *CI* [−0.002, 0.099]); from Spain and California (MgRi–HgtRf; *F*_ST_ = 0.042; *CI* [−0.005, 0.092]) or from this latter and Chile (HgtRf–MgDi; *F*_ST_ = 0.025; *CI* [−0.013, −0.072] (Table S4). The differentiation parameter *D*_EST_ ranged between 0.013 (MgYo–MgNo) and 0.450 (MgNo–MgNd) and averaged 0.299 ± 0.110 between samples of *M. galloprovincialis*. This parameter was significant between all pairwise comparisons, except between samples from the same region, i.e., (MgYo–MgNo; *D*_EST_ = 0.013; *CI* [−0.019, 0.067]) and (MgtRf–MgtMb; *D*_EST_ = 0.066; *CI* [−0.015, 0.191]) (Table S4). Both parameters correlated positively with each other (*y =* 1.5344*x +* 0.1689, *r*^2^ = 0.7355).

**Figure 2:**
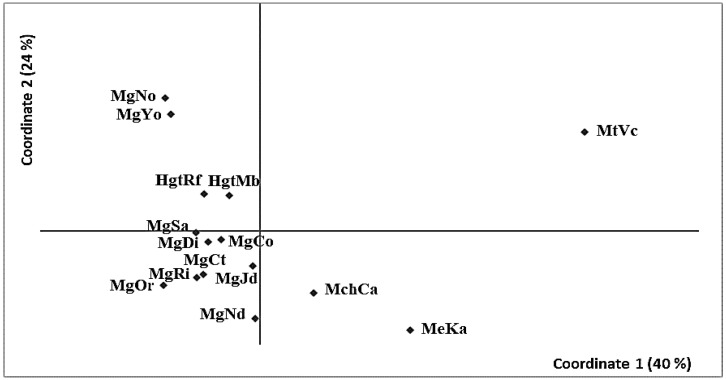
Principal coordinates analysis on the genetic distance *F*_ST_ between samples of *M. galloprovincialis* (Mg) relative to the control species (Mch, *M. chilensis*; Me, *M. edulis*; Mt, *M. trossulus*).

The first pCoA explained 40% of the divergence between samples and separated unambiguously the four species comprised in the analysis (Figure 2). *M. trossulus* was the most divergent sample. The largest interspecific variance (as averaged among all pairwise comparisons) was observed between *M. trossulus* and *M. galloprovincialis* [*F*_ST_(Mt–Mg) = 0.312 ± 0.037, *p* = 0.0019] as compared to *M. edulis* [*F*_ST_(Mt–Me) = 0.249] and to *M. chilensis* [*F*_ST_(Mt–Mch) = 0.259]. The second pCoA coordinate explained 24% of the variation and showed divergence within *M. galloprovincialis*, such as the two Japanese samples (MgYo, MgNo) or the two samples from the *M. galloprovincialis*–*M. trossulus* hybrid zone in California (MgtRf, MgtMb).

**Figure 3:**
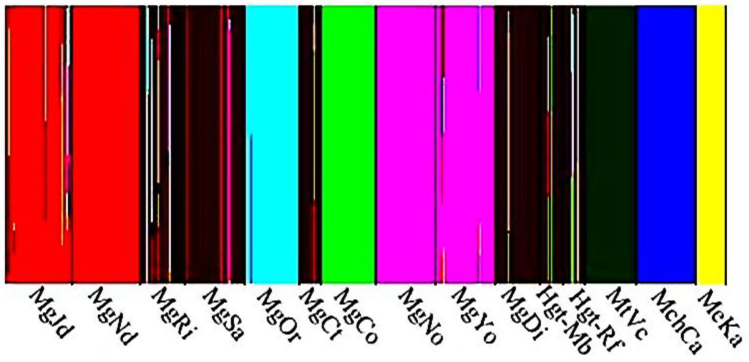
BAPS posterior probability of samples of belonging to one of the five gene pools within *M. galloprovincialis* (Mg), to *M. trossulus* (Mt, dark green), to *M. chilensis* (Mch, dark blue) and to *M. edulis* (Me, yellow). Only the significant (*alpha* = 0.05) admixture estimates are shown.

The significant admixture estimates from the Bayesian clustering inferred by BAPS showed one gene pool for each species involved in the analysis. Five gene pools were significant within *M. galloprovincialis* (Figure 3). The largest gene pool includedsix samples, i.e., North Atlantic Spanish (MgRi and MgSa), South Africa (MgCt), Chile (MgDi) and California (HgtMb and HgtRf). The most heterogeneous pool composition was observed in sample MgRi from Galicia (Spain) and in the two Californian samples. The other four pools from Morocco, South Africa, Australia and Japan showed less admixture.

**Figure 4:**
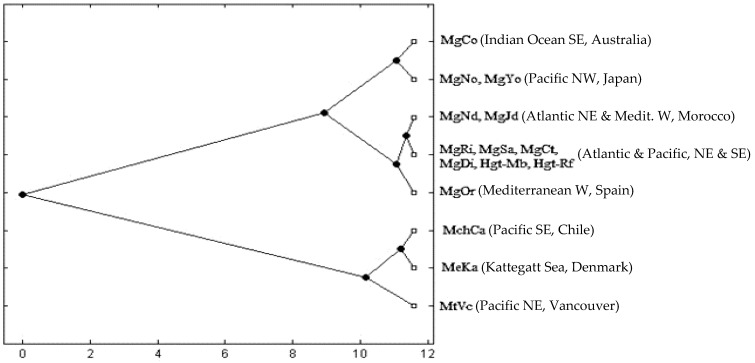
BAPS reconstruction relating eight significant sample clusters upon a neighbor-joining dendrogram based on the Nei’s distance (averaged over loci).

The highest divergence between the *k* = 8 BAPS pools using the Nei genetic distance was observed between *M. galloprovincialis* and the rest of the species, i.e., *M. trossulus* (1.152), *M. edulis* (0.657) and *M. chilensis* (0.506). The *M. galloprovincialis* samples formed a clade that was the sister group of the rest of the species. The clade of *M. galloprovincialis* included two subclades, one grouping the East Asia samples (Japan and Australia) and the other joining all the Atlantic North samples of Iberia and Morocco and including South Africa, Chile and California (Figure 4).

The fixation indexes within *M. galloprovincialis* showed a high genetic variation between samples (*F*_ST_ = 0.096*). Such variation was higher within large continental regions (*F*_CT_ = 0.058, *p* = 0.0078) than among regions (*F*_CT_ = 0.046, *p* = 0.0089), although both were significant (Table 2). No variation was observed between hemispheres (*F*_CT_ = 0.007, *p* = 0.258). The largest interspecific variance was observed between *M. galloprovincialis* and *M. trossulus* (*F*_CT_ = 0.230, *p* = 0.0019) as compared to other pairwise comparisons.

## 4. Discussion

### 4.1. Genetic Diversity of M. galloprovincialis

The degree of interspecific conservation of microsatellite primers and their polymorphism were proportional to the genetic distance between species [44]. Herein, all primer pairs from *M. galloprovincialis* were not only amplified in the sample of *M. chilensis* but this latter bore a significantly higher allelic richness and number of specific alleles than the former species. This phenomenon points to both the similarity of these genomes and their own specific status [59]. The average polymorphism of seven microsatellites of *M. galloprovincialis* from all the continents (mean *H*_E_ *± SD* = 0.723 ± 0.183) was congruent with previous observations in this species, e.g., *H*_E_ = 0.772 ± 0.154 from six microsatellites on Iberian samples [26], but slightly higher than in seven microsatellites from the Moroccan samples (*H*_E_ = 0.552 ± 0.127) [22]. This result was expected because of both the sampling amplitude and the population size, i.e., the genetic diversity is maximal in Galician Estuaries (Rías Gallegas) inhabited by the largest world population of this species [31]. The global deficit of heterozygotes, especially at loci *Mg*µ1 and *Mg*µ2, indicated a significant deviation from the Hardy–Weinberg equilibrium. This phenomenon was reported earlier in microsatellites of *M. galloprovincialis* [22,26] but was also apparent with allozymes and nuclear DNA markers [2,60] and is common in marine bivalves [61,62]. The underlying causes of that deficit range from functional to technical. Some functional hypotheses are selection [63,64], stock admixture [65], inbreeding and genotype-independent spawning [66]. More likely, technical causes are stochastic genotyping errors [67] such as allelic dropout or false alleles [68], as well as systematic errors, i.e., null alleles due to primer site sequence variation [69]. The high putative null allele frequency (~15%) inferred in this study for two loci across most samples is a reasonable explanation for their heterozygote deficit. The other loci showed a low or moderate null allele frequency (below 10%), but the analytical exclusion of loci *Mg*µ1 and *Mg*µ2 did not produce a different outcome regarding genetic diversity, as observed in most studies [70].

### 4.2. Genetic Differentiation between Species

The analysis of principal coordinates separated the four species comprised in the analysis, with *M. trossulus* as the most distinct taxon of the genus, as shown using DNA, allozyme and morphometrics markers, e.g., [71]. *M. trossulus* is more distantly related to *M. galloprovincialis* than to *M. edulis*, i.e., average *F*_ST_ (Mt–Mg) = 0.312 ± 0.037, *p* = 0.001 and *F*_ST_ (Mt–Me) = 0.249, *p* = 0.001, respectively, and is believed to have been diverging from these species for about 3.5 million years [72]. The interspecific relationships upon variance components are congruent with the Nei genetic distance computed after the *k* = 8 BAPS pools, where *M. galloprovincialis* diverged from the rest of species—*M. trossulus*, *M. edulis* and *M. chilensis*—by 1.152, 0.657 and 0.506, respectively. Previous studies situated *M. galloprovincialis* and *M. edulis* as the most closely related sister taxa of this genus, which coalesced some 2 million years ago [73]. However, the present scenario suggests that *M. chilensis* is an intermediate taxon between *M. galloprovincialis* and *M. edulis* but closer to the former, as previously shown with allozymes [59], microsatellites [44] and mtDNA COI [74] (but also see mitogenomic analyses [75]). These population genetic studies on *M. chilensis* and its latitudinal morphological description [76] provided the first proofs of the specific genetic status of this taxon in Chile. Despite the geographic proximity and hybridization between *M. chilensis* and *M. edulis platensis* in Cape Horn [77], *M. chilensis* seems to be genetically closer to *M. galloprovincialis*. That similarity could be phylogenetic rather than introgressive because of both the assumed recent introduction of *M. galloprovincialis* in central Chile [78,79,80] and its lack of admixture with *M. chilensis* [77].

### 4.3. Genetic Differentiation within M. galloprovincialis

The amount of interpopulation genetic divergence in *M. galloprovincialis* observed with seven microsatellites (*F*_ST_ = 0.102 ± 0.044) was slightly higher than that reported from thousands of SNP markers (*F*_ST_ = 0.087, [18]) and is likely due to an overestimation of null alleles [70]. This result shows that a moderate number of random microsatellites provide enough signal for global population genetic analysis. None of the five regional gene pools identified within *M. galloprovincialis* using Bayesian clustering was explained by divergence between hemispheres, as has also been reported upon COI gene sequences [40], but better by variation within regions and among regions. The variation within regions is the byproduct of a population connectivity pattern reported in many instances between samples of Northern Africa (Morocco) and Mediterranean coasts, e.g., *F*_ST_ = 0.044 ± 0.006 [22], or between Atlantic, Alboran and Mediterranean Iberian coasts (*F*_CT_ = 0.0281, *p* = 0.023; [26]). Notably, within-region divergence equaled that among regions, although the latter was expected to be much higher under an isolation-by-distance pattern and independent regional evolution. Except for the Australian and New Zealand samples, the global scenario suggests that the *M. galloprovincialis* populations inhabiting exotic locations are relatively recently settled. The small genetic divergence (*F*_ST_) and genetic differentiation (*D*_EST_) between close samples, i.e., Japanese or California samples, as well as the high divergence between distant samples, e.g., Mediterranean vs. Australian samples, are observations congruent with previous studies using allozymes [81] and DNA markers [78].

### 4.4. Patterns of Divergence in Parapatry

Although the majority of pairwise comparisons were significant for both *D*_EST_ and *F*_ST_, the higher conservativism of *F*_ST_ identified a lack of divergence between very distant samples such as North Spain (MgRi) and both South Africa (MgCt) and California (MgtRf) or between this latter and Chile (MgDi). That unexpected genetic similarity was patent in two major subclades within *M. galloprovincialis*, i.e., one grouping being East Asia samples (Japan and Australia) and the other grouping being all Atlantic North samples including South Africa, Chile and California in both a single tree subclade and a single gene pool. These results suggests that the global genetic scenario of *M. galloprovincialis* is composed by two major patterns. One pattern comprises those regions diverging evolutionarily in parapatry, e.g., Iberian MgRi (Atlantic) vs. MgOr (Mediterranean) separated by the Almería-Oran Oceanographic Front [82], where strong congruence exists between genetic markers, e.g., allozymes, mtDNA and microsatellites [22,27,81,83,84,85]. A second pattern is shown between distant populations that did not diverge in parapatry but had been recently segregated either from a donor population or from one of its exotic introduction sites. Such recent settlements are understood as accidental through intercontinental trading, e.g., in ballast water or hull foiling [86,87,88], or by aquaculture interests, whether aware or not of the consequences of biological translocations. For instance, there has been much investigation on the invasive capacity of *M. galloprovincialis* in South Africa, California and Chile after the colonization event of this species in the last century [89,90,91,92]. Current data suggest that Atlantic Southwestern Europe is the direct or indirect source of present-day populations in California, South Africa and Chile. Supporting this suggested Atlantic origin is that the largest distance observed between the Mediterranean sample MgOr and the Japanese sample MgYo was even higher (*F*_ST_ = 0.186) than that observed between species, e.g., *M. galloprovincialis*–*M. chilensis* (0.116 ± 0.043). This hypothesis of an Atlantic Northeast origin of South Africa mussels is also supported by haplotype networks and *F*_ST_ data from mtDNA analyses, although not recognized as such therein [40]. Nevertheless, the Atlantic origin hypothesis disagrees with most previous works supporting a single Mediterranean origin of exotic *M. galloprovincialis*, as claimed using SNPs markers on the Chilean [18,93] or Brazilian samples [36] of *M. galloprovincialis*.

### 4.5. The Pacific Northeast

The genetic status of *M. galloprovincialis* from the hybrid zone of California (HgtMb and HgtRf) is congruent with its historical lack of introgression with native *M. trossulus* [94]. This genetic status of Californian *M. galloprovincialis* agrees with previous studies in the Pacific Northeast from Puget Sound to the central California hybrid zone [95], as well as with the interspecific polarized distribution in the latter region, e.g., Morro Bay [35]. Japan and southern Europe have been suggested as putative donors of the multiple introductions suspected to have occurred in the Pacific Northeast [89]. The European origin was Mediterranean from analyses of allozyme data [9,81,96] and genomic DNA [78]. However, as indicated above for other exotic locations, the Atlantic North European population is the most likely origin of those Pacific settlements, according to current microsatellites.

### 4.6. The Pacific Southeast

The *Mytilus* species inhabiting the Pacific Southeast is *M. chilensis* [97], which has been shown to be a genetically distinct taxon in the last decade [44,74]. *M. galloprovincialis* is also present in the Chilean coast [10,79,98], and, to date, no evidence exists on either its expansion beyond the Gulf of Arauco in the Bío-Bío Region or its hybridization with the native *M. chilensis* [77]. Nonetheless, this latter naturally hybridizes with the neighboring species *M. edulis platensis* in the Southern Cone tip [77]. The natural Pacific east occurrence of *Mytilus*-like fossils in South America [99], as well as those in North America [9,89,94,100], does not help clarify a putative trans-equatorial historical migration of *Mytilus* and other taxa between these two subcontinents [101]. Present knowledge allows for thesuggestion that the Pacific coasts were originally occupied by distinct species, i.e., *M. trossulus* or its predecessor in the Pacific north, *M. californianus* [102] and *M. chilensis* or a putative predecessor in the Pacific south and*M. galloprovincialis* in the Southern Hemisphere [3]. Also, *M. galloprovincialis* seems to have been introduced multiple times to the Pacific north, via Japan or Europe [89]. However, the assumed source origin of Chilean *M. galloprovincialis* in the Mediterranean [78], is not supported by current microsatellite data. Because of the genetic similarity after Bayesian analyses (see Figure 4) between Northern Hemisphere *M. galloprovincialis* and the Chilean mussel from Dichato, the origin of this latter appears to be California, Atlantic Europe or Cape town. For instance, given that phylogeographic evidence exists on the accidental introduction of *M. galloprovincialis* to South Africa [103], the Pacific Southeast population could have its origin in the native distribution area and/or in one of its exotic settlements.

### 4.7. Australia and Japan

A historical circum-Arctic migration from Atlantic European coasts has been reported to explain the existence of mussels in Australia [2,3,104]. A trans-equatorial migration of mussels between the North and the South Pacific has also been proposed to explain their occurrence in those regions [81]. These hypotheses are not mutually exclusive because a first circum-artic migration could have reached the Pacific Northwest and been followed by a trans-equatorial migration to Australia. In addition, knowledge on copepod parasitism of Japanese mussels [105] suggests a relatively recent human introduction of European mussels into Japanese coasts [106], likely during the Edo period of Japanese history. Whatever hypothesis is correct, the native range of *M. galloprovincialis* about 1 My ago would include Australia and New Zealand and possibly Chile [1,107,108,109]. Advancing in time, local evolution in parapatry and/or new introductions would have produced actual representatives of this genus, such as Southern-Hemisphere *M. galloprovincialis* [110] and *M. chilensis* [97], respectively.

Multiple introductions of *M. galloprovincialis* into Australia and New Zealand from its Atlantic and Mediterranean natural range have been suggested based on genetic and demographic data [111]. Those introductions have led to admixtures with the native *Mytilus planulatus* over a large amount of the Australian coastline [112], which can explain previous scenarios of genetic heterogeneity of *Mytilus* samples from Australia [108,109]. Current analyses indicate that the two mussel samples from Australia and Japan belong to Northern-Hemisphere *M. galloprovincialis,* yet they are highly divergent from each other, as well as from the rest of the gene pools of this species (see Figure 4), as has also been reported in COI sequence data [40]. The inter-cluster divergence within *M. galloprovincialis* suggests a common origin of those two samples, while their intra-cluster divergence suggests a younger divergence between them. The above hypotheses on population sourcing from Europe to Japan or to Australia, and then from Japan to Australia or vice versa, can reasonably explain the current parapatric scenario observed with microsatellites.

## 5. Conclusions

Despite the high genetic variation exhibited by *M. galloprovincialis*, it maintains its genetic integrity on a global scale. Microsatellite variation confirms the higher divergence of *M. trossulus* from its congeneric species and places *M. chilensis* as an intermediate taxon between *M. galloprovincialis* and *M. edulis*. Also, microsatellite variation identifies *M. galloprovincialis* from the Atlantic Northeast as the most likely source of exotic settlements worldwide. The adaptive potential of *M. galloprovincialis* allows it to be considered a flexible evolutionary species (FES), i.e., a species or population whose genetic background allows it to adapt rapidly to changing environments. The plastic adaptation of this species makes it a resilient candidate to counteract stressful conditions and provide ecological opportunities to many intertidal genera facing global coastal rarefaction.

## Figures and Tables

**Table 1:** Sampling locations and working codes for 15 samples of *Mytilus* spp. (*M. galloprovincialis*, *M. chilensis*, putative hybrids *M. trossulus*–*M. galloprovincialis* and *M. edulis*) analyzed in this study.

Species	Ocean	Location	Code	Size	Coordinates
*M. galloprovincialis*	Atlantic Northeast (Spain)	Ribeira	MgRi	40	42°32′ N/08°59′ W
		Sanxenxo	MgSa	40	42°23′ N/08°48′ W
	Mediterranean West (Spain)	Oropesa	MgOr	37	40°08′ N/00°15′ E
	Alboran Sea (Morocco) Atlantic Northeast (Morocco)	Nador	MgNd	45	35°39’ N/03°03’ W
		El Jadida	MgJd	45	32°39′ N/08°51′ W
	Atlantic Southeast (South Africa)	Cape Town	MgCt	14	33°54′ S/18°27′ E
	Pacific Southeast (Chile)	Dichato	MgDi	30	36°32′ S/72°47′ W
	Pacific Northwest (Japan)	Nojima	MgNo	40	32°59′ N/135°21′ E
		Yokohama	MgYo	40	35°25′ N/139°39′ E
	Indian Southeast (Australia)	Cockburn Sound	MgCo	37	32°10′ S/115°43′ E
*M. galloprovincialis–M. trossulus*	Pacific Northeast (USA)	California	HgtMb	15	35°21′ N/120°51′ W
			HgtRf	15	37°57′ N/122°29′ W
*M. chilensis*	Pacific Southeast (Chile)	Caicaen	MchCa	40	41°47′ S/73°10′ W
*M. edulis*	Atlantic Northeast (Denmark)	Kattegat	MeKa	20	56°08′ N/10°14′ E
*M. trossulus*	Pacific Northeast (Canada)	Vancouver	MtVc	34	49°16′ N/123°10′ W

**Table 2:** Hierarchical analysis of molecular variance (AMOVA) scored at different geographic and taxonomic levels in *Mytilus* spp. Asterisks indicate the probability, based on 1023 permutations, that the observed values were equal to or smaller than that expected by random is *p* ≤ 0.01; ns: not significant.

Source of Variation	d.f.	Sum of Squares	Variance Components	Percentage of Variation	Fixation Indexes
*M. galloprovincialis*					
Among samples	11	216.636	0.259	9.64	*F*_ST_ = 0.096 *
Among individuals within samples	376	1153.357	0.630	23.37	*F*_IS_ = 0.258 *
Within individuals	388	701.000	0.630	66.99	*F*_IT_ = 0.330 *
Total	775	2070.994	2.696		
*M. galloprovincialis* (regional differentiation)					
Among groups	5	141.122	0.125	4.61	*F*_CT_ = 0.046 *
Among samples within groups	6	75.514	0.151	5.57	*F*_SC_ = 0.058 *
Among individuals within samples	376	1153.357	0.630	23.23	*F*_IS_ = 0.258 *
Within individuals	388	701.000	1.806	66.59	*F*_IT_ = 0.334 *
*M. galloprovincialis* (North vs. South Hemispheres)					
Among groups	1	24.11	0.019	0.70	*F*_CT_ = 0.007 ^ns^
Among samples within groups	10	192.526	0.252	9.34	*F*_SC_ = 0.094 *
Among individuals within samples	376	1153.357	0.630	23.27	*F*_IS_ = 0.258 *
Within individuals	388	701.000	1.806	66.69	*F*_IT_ = 0.333 *
*M. galloprovincialis* vs. (*M. chilensis, M. edulis* and *M. trossulus*)					
Among groups	1	83.690	0.197	6.82	*F*_CT_ = 0.068 *
Among samples within groups	13	293.075	0.307	10.64	*F*_SC_ = 0.114 *
Among individuals within samples	467	1414.498	0.642	22.22	*F*_IS_ = 0.269 *
Within individuals	482	840.500	1.743	60.32	*F*_IT_ = 0.396 *
*M. galloprovincialis* vs. *M. chilensis*					
Among groups	1	60.988	0.150	5.25	*F*_CT_ = 0.052 *
Among samples within groups	12	216.636	0.228	7.98	*F*_SC_ = 0.084 *
Among individuals within samples	454	1426.857	0.659	23.02	*F*_IS_ = 0.265 *
Within individuals	468	854.000	1.824	63.75	*F*_IT_ = 0.362 *
*M. galloprovincialis* vs. *M. edulis*					
Among groups	1	52.292	0.267	9.19	*F*_CT_ = 0.091 *
Among samples within groups	12	216.636	0.242	8.33	*F*_SC_ = 0.091 *
Among individuals within samples	414	1250.757	0.618	21.21	*F*_IS_ = 0.257 *
Within individuals	428	764.000	1.785	61.27	*F*_IT_ = 0.387 *
*M. galloprovincialis* vs. *M. trossulus*					
Among groups	1	195.329	0.761	23.01	*F*_CT_ = 0.230 *
Among samples within groups	12	216.636	0.234	7.09	*F*_SC_ = 0.092 *
Among individuals within samples	442	1304.740	0.638	19.28	*F*_IS_ = 0.275 *
Within individuals	456	764.000	1.675	50.62	*F*_IT_ = 0.493 *
Total variance					
Among samples	14	376.765	0.374	13.57	*F*_ST_ = 0.135 *
Within samples	467	1414.498	0.642	23.27	*F*_IS_ = 0.269 *
Within individuals	482	840.500	1.743	63.16	*F*_IT_ = 0.368 *
Total	963	2631.763	2.761		

## Data Availability

All data generated is contained either in the article or in the Supplementary Materials.

## Supplementary Materials

The following supporting information can be downloaded at https://www.mdpi.com/article/10.3390/ani13243754/s1, Table S1: Allelic frequencies of seven microsatellites analyzed in 15 samples of *Mytilus* spp. (sample codes are given in Table 1); Table S2: Genetic diversity of seven microsatellites in twelve samples of *M. galloprovincialis*, three samples of congeneric species (*M. trossulus* (MtVc), *M. chilensis* (MchCA) and *M. edulis* (MeKa) and two samples from a *M. galloprovincialis*–*M. trossulus* hybrid zone (HgtMb and HgtRf); Table S3: Pairwise estimates of genetic differentiation (*D*_EST_, above diagonal) and gene distance (*F*_ST_, below diagonal) between samples of *Mytilus* spp. All values except those bolded were significantly different from zero. The significance of *D*_EST_ was drawn from its 95% confidence interval (Table S4). The significance threshold for *F*_ST_ was generated after 100 MC batches of 5000 iterations each for *alpha* = 0.01; Table S4: Confidence intervals (95%) for *D*_EST_ (above diagonal) and *F*_ST_ (below diagonal).
