# Supplementary material for: Population Genetic Divergence among Worldwide Gene Pools of the Mediterranean Mussel Mytilus galloprovincialis
Source: Animals (Basel). 2023 Dec 5;13(24):3754. doi: 10.3390/ani13243754 (PMC10740466; doi:10.3390/ani13243754)
Supplement: Supplementary file 1 [file animals-13-03754-s001.zip › animals-2713593-supplementary.pdf]

Table S1. Allelic frequencies of seven microsatellites analysed in 15 samples of *Mytilus* spp. (sample codes are given in **Table 1**).

Locus	Allele	MgJd	MgNd	MgRi	MgSa	MgOr	MgCt	MgCo	MgNo	MgYo	MgDi	HgtMb	HgtRf	MtVc	MchCa	MeKa
***Mg*μ1**	120	0.012	0.000	0.000	0.000	0.000	0.000	0.000	0.000	0.000	0.000	0.000	0.000	0.000	0.000	0.000
	122	0.000	0.000	0.000	0.000	0.000	0.000	0.000	0.000	0.000	0.000	0.036	0.000	0.000	0.000	0.000
	124	0.000	0.000	0.000	0.000	0.000	0.000	0.472	0.052	0.034	0.050	0.036	0.033	0.000	0.088	0.000
	126	0.000	0.000	0.000	0.000	0.000	0.000	0.042	0.121	0.000	0.050	0.143	0.367	0.000	0.063	0.000
	128	0.000	0.000	0.000	0.000	0.000	0.000	0.069	0.000	0.000	0.000	0.000	0.033	0.000	0.000	0.000
	130	0.083	0.100	0.050	0.000	0.041	0.000	0.347	0.190	0.241	0.117	0.321	0.167	0.000	0.050	0.000
	132	0.000	0.011	0.050	0.064	0.000	0.071	0.014	0.155	0.155	0.017	0.000	0.100	0.000	0.000	0.000
	134	0.000	0.011	0.000	0.000	0.027	0.000	0.028	0.000	0.000	0.017	0.000	0.033	0.000	0.000	0.000
	136	0.000	0.000	0.000	0.000	0.000	0.000	0.000	0.000	0.000	0.083	0.000	0.000	0.000	0.000	0.000
	137	0.262	0.233	0.050	0.026	0.081	0.071	0.000	0.017	0.052	0.050	0.179	0.067	0.000	0.000	0.000
	138	0.000	0.078	0.117	0.090	0.176	0.071	0.014	0.121	0.052	0.033	0.036	0.000	0.000	0.013	0.000
	140	0.024	0.033	0.133	0.013	0.270	0.000	0.014	0.000	0.017	0.150	0.179	0.133	0.000	0.000	0.000
	142	0.012	0.000	0.467	0.705	0.108	0.536	0.000	0.259	0.362	0.317	0.036	0.067	0.000	0.000	0.000
	146	0.167	0.211	0.083	0.051	0.000	0.250	0.000	0.069	0.069	0.117	0.036	0.000	1.000	0.150	1.000
	148	0.000	0.022	0.017	0.026	0.000	0.000	0.000	0.017	0.017	0.000	0.000	0.000	0.000	0.025	0.000
	150	0.012	0.022	0.017	0.013	0.014	0.000	0.000	0.000	0.000	0.000	0.000	0.000	0.000	0.013	0.000
	152	0.012	0.011	0.000	0.000	0.000	0.000	0.000	0.000	0.000	0.000	0.000	0.000	0.000	0.125	0.000
	154	0.071	0.011	0.000	0.013	0.095	0.000	0.000	0.000	0.000	0.000	0.000	0.000	0.000	0.075	0.000
	156	0.012	0.022	0.017	0.000	0.054	0.000	0.000	0.000	0.000	0.000	0.000	0.000	0.000	0.013	0.000
	158	0.179	0.044	0.000	0.000	0.041	0.000	0.000	0.000	0.000	0.000	0.000	0.000	0.000	0.075	0.000
	160	0.012	0.000	0.000	0.000	0.068	0.000	0.000	0.000	0.000	0.000	0.000	0.000	0.000	0.063	0.000
	162	0.012	0.044	0.000	0.000	0.014	0.000	0.000	0.000	0.000	0.000	0.000	0.000	0.000	0.013	0.000
	164	0.071	0.078	0.000	0.000	0.000	0.000	0.000	0.000	0.000	0.000	0.000	0.000	0.000	0.013	0.000
	166	0.000	0.011	0.000	0.000	0.000	0.000	0.000	0.000	0.000	0.000	0.000	0.000	0.000	0.000	0.000
	168	0.024	0.044	0.000	0.000	0.000	0.000	0.000	0.000	0.000	0.000	0.000	0.000	0.000	0.050	0.000
	170	0.036	0.000	0.000	0.000	0.000	0.000	0.000	0.000	0.000	0.000	0.000	0.000	0.000	0.088	0.000
	172	0.000	0.000	0.000	0.000	0.014	0.000	0.000	0.000	0.000	0.000	0.000	0.000	0.000	0.000	0.000
	174	0.000	0.011	0.000	0.000	0.000	0.000	0.000	0.000	0.000	0.000	0.000	0.000	0.000	0.088	0.000
***Mg*μ2**	135	0.000	0.000	0.000	0.000	0.000	0.000	0.027	0.000	0.000	0.000	0.000	0.000	0.000	0.000	0.000
	155	0.011	0.000	0.000	0.000	0.000	0.000	0.000	0.026	0.000	0.017	0.000	0.000	0.000	0.000	0.000
	159	0.000	0.000	0.000	0.000	0.000	0.000	0.000	0.000	0.025	0.000	0.000	0.167	0.000	0.000	0.000
	161	0.000	0.011	0.000	0.000	0.000	0.000	0.014	0.000	0.000	0.017	0.036	0.083	0.000	0.000	0.000
	163	0.011	0.178	0.050	0.000	0.000	0.000	0.000	0.039	0.038	0.017	0.000	0.083	0.000	0.000	0.000
	165	0.200	0.300	0.217	0.288	0.014	0.179	0.189	0.408	0.438	0.183	0.214	0.125	0.000	0.000	0.000
	167	0.233	0.078	0.233	0.163	0.838	0.286	0.230	0.276	0.213	0.467	0.250	0.458	0.000	0.000	0.000
	169	0.122	0.056	0.150	0.038	0.081	0.036	0.068	0.013	0.025	0.000	0.036	0.042	0.000	0.000	0.000
	171	0.078	0.022	0.050	0.163	0.041	0.143	0.162	0.053	0.100	0.033	0.000	0.042	0.000	0.000	0.000
	173	0.089	0.033	0.000	0.013	0.000	0.179	0.216	0.026	0.075	0.167	0.036	0.000	0.000	0.000	0.000
	175	0.044	0.022	0.017	0.050	0.014	0.071	0.000	0.026	0.000	0.067	0.000	0.000	0.000	0.000	0.000
	177	0.011	0.044	0.000	0.038	0.014	0.000	0.081	0.053	0.000	0.000	0.036	0.000	0.000	0.000	0.000
	179	0.000	0.000	0.050	0.000	0.000	0.000	0.000	0.039	0.000	0.017	0.143	0.000	0.000	0.000	0.036
	181	0.022	0.011	0.000	0.000	0.000	0.000	0.000	0.000	0.013	0.000	0.179	0.000	0.000	0.026	0.000
	183	0.011	0.011	0.033	0.000	0.000	0.071	0.014	0.026	0.000	0.000	0.036	0.000	0.000	0.000	0.000
	185	0.000	0.033	0.017	0.038	0.000	0.000	0.000	0.000	0.013	0.000	0.000	0.000	0.000	0.000	0.000
	187	0.000	0.022	0.033	0.038	0.000	0.036	0.000	0.013	0.038	0.000	0.000	0.000	0.000	0.000	0.000
	189	0.056	0.000	0.050	0.000	0.000	0.000	0.000	0.000	0.025	0.000	0.000	0.000	0.000	0.064	0.107
	191	0.000	0.011	0.000	0.050	0.000	0.000	0.000	0.000	0.000	0.000	0.036	0.000	0.000	0.038	0.036
	193	0.011	0.022	0.017	0.025	0.000	0.000	0.000	0.000	0.000	0.000	0.000	0.000	0.000	0.051	0.000
	195	0.000	0.011	0.033	0.013	0.000	0.000	0.000	0.000	0.000	0.000	0.000	0.000	0.000	0.064	0.000
	197	0.000	0.000	0.000	0.000	0.000	0.000	0.000	0.000	0.000	0.017	0.000	0.000	0.000	0.013	0.071
	199	0.011	0.022	0.000	0.000	0.000	0.000	0.000	0.000	0.000	0.000	0.000	0.000	0.000	0.000	0.071
	201	0.022	0.022	0.000	0.050	0.000	0.000	0.000	0.000	0.000	0.000	0.000	0.000	0.000	0.000	0.071
	203	0.000	0.000	0.000	0.000	0.000	0.000	0.000	0.000	0.000	0.000	0.000	0.000	0.000	0.026	0.000
	205	0.022	0.000	0.000	0.000	0.000	0.000	0.000	0.000	0.000	0.000	0.000	0.000	1.000	0.013	0.036

Supplementary table 1, cont.

Locus	Allele	MgJd	MgNd	MgRi	MgSa	MgOr	MgCt	MgCo	MgNo	MgYo	MgDi	H-MB	H-RF	McVc	MchCa	MeKa
	207	0.011	0.011	0.000	0.013	0.000	0.000	0.000	0.000	0.000	0.000	0.000	0.000	0.000	0.026	0.071
	209	0.000	0.011	0.000	0.000	0.000	0.000	0.000	0.000	0.000	0.000	0.000	0.000	0.000	0.000	0.000
	211	0.000	0.000	0.017	0.000	0.000	0.000	0.000	0.000	0.000	0.000	0.000	0.000	0.000	0.026	0.143
	213	0.011	0.022	0.017	0.013	0.000	0.000	0.000	0.000	0.000	0.000	0.000	0.000	0.000	0.051	0.000
	215	0.000	0.011	0.000	0.000	0.000	0.000	0.000	0.000	0.000	0.000	0.000	0.000	0.000	0.038	0.036
	217	0.022	0.000	0.000	0.000	0.000	0.000	0.000	0.000	0.000	0.000	0.000	0.000	0.000	0.013	0.000
	219	0.000	0.000	0.000	0.013	0.000	0.000	0.000	0.000	0.000	0.000	0.000	0.000	0.000	0.026	0.036
	221	0.000	0.000	0.000	0.000	0.000	0.000	0.000	0.000	0.000	0.000	0.000	0.000	0.000	0.026	0.000
	223	0.000	0.000	0.017	0.000	0.000	0.000	0.000	0.000	0.000	0.000	0.000	0.000	0.000	0.000	0.000
	225	0.000	0.022	0.000	0.000	0.000	0.000	0.000	0.000	0.000	0.000	0.000	0.000	0.000	0.026	0.071
	227	0.000	0.000	0.000	0.000	0.000	0.000	0.000	0.000	0.000	0.000	0.000	0.000	0.000	0.013	0.179
	229	0.000	0.011	0.000	0.000	0.000	0.000	0.000	0.000	0.000	0.000	0.000	0.000	0.000	0.051	0.000
	231	0.000	0.000	0.000	0.000	0.000	0.000	0.000	0.000	0.000	0.000	0.000	0.000	0.000	0.013	0.000
	235	0.000	0.000	0.000	0.000	0.000	0.000	0.000	0.000	0.000	0.000	0.000	0.000	0.000	0.026	0.000
	247	0.000	0.000	0.000	0.000	0.000	0.000	0.000	0.000	0.000	0.000	0.000	0.000	0.000	0.026	0.000
	253	0.000	0.000	0.000	0.000	0.000	0.000	0.000	0.000	0.000	0.000	0.000	0.000	0.000	0.038	0.000
	257	0.000	0.000	0.000	0.000	0.000	0.000	0.000	0.000	0.000	0.000	0.000	0.000	0.000	0.038	0.000
	263	0.000	0.000	0.000	0.000	0.000	0.000	0.000	0.000	0.000	0.000	0.000	0.000	0.000	0.013	0.036
	265	0.000	0.000	0.000	0.000	0.000	0.000	0.000	0.000	0.000	0.000	0.000	0.000	0.000	0.026	0.000
	267	0.000	0.000	0.000	0.000	0.000	0.000	0.000	0.000	0.000	0.000	0.000	0.000	0.000	0.038	0.000
	269	0.000	0.000	0.000	0.000	0.000	0.000	0.000	0.000	0.000	0.000	0.000	0.000	0.000	0.038	0.000
	273	0.000	0.000	0.000	0.000	0.000	0.000	0.000	0.000	0.000	0.000	0.000	0.000	0.000	0.026	0.000
	277	0.000	0.000	0.000	0.000	0.000	0.000	0.000	0.000	0.000	0.000	0.000	0.000	0.000	0.013	0.000
	281	0.000	0.000	0.000	0.000	0.000	0.000	0.000	0.000	0.000	0.000	0.000	0.000	0.000	0.038	0.000
	283	0.000	0.000	0.000	0.000	0.000	0.000	0.000	0.000	0.000	0.000	0.000	0.000	0.000	0.013	0.000
	287	0.000	0.000	0.000	0.000	0.000	0.000	0.000	0.000	0.000	0.000	0.000	0.000	0.000	0.013	0.000
	291	0.000	0.000	0.000	0.000	0.000	0.000	0.000	0.000	0.000	0.000	0.000	0.000	0.000	0.026	0.000
	293	0.000	0.000	0.000	0.000	0.000	0.000	0.000	0.000	0.000	0.000	0.000	0.000	0.000	0.013	0.000
	295	0.000	0.000	0.000	0.000	0.000	0.000	0.000	0.000	0.000	0.000	0.000	0.000	0.000	0.013	0.000
*Mg*μ3	132	0.011	0.022	0.033	0.000	0.000	0.000	0.014	0.000	0.000	0.017	0.000	0.000	0.221	0.000	0.000
	134	0.000	0.011	0.000	0.000	0.000	0.000	0.000	0.000	0.000	0.000	0.000	0.000	0.162	0.000	0.000
	136	0.056	0.000	0.017	0.000	0.000	0.036	0.056	0.000	0.000	0.000	0.000	0.000	0.118	0.000	0.000
	138	0.467	0.556	0.450	0.500	0.865	0.571	0.292	0.750	0.730	0.567	0.567	0.567	0.132	0.238	0.425
	139	0.000	0.000	0.000	0.000	0.000	0.000	0.097	0.000	0.000	0.000	0.000	0.000	0.000	0.000	0.000
	140	0.389	0.400	0.400	0.425	0.135	0.393	0.528	0.250	0.270	0.317	0.433	0.333	0.338	0.738	0.550
	141	0.000	0.000	0.033	0.038	0.000	0.000	0.000	0.000	0.000	0.000	0.000	0.000	0.000	0.000	0.000
	142	0.078	0.011	0.067	0.025	0.000	0.000	0.014	0.000	0.000	0.100	0.000	0.100	0.000	0.013	0.000
	144	0.000	0.000	0.000	0.013	0.000	0.000	0.000	0.000	0.000	0.000	0.000	0.000	0.029	0.000	0.025
	146	0.000	0.000	0.000	0.000	0.000	0.000	0.000	0.000	0.000	0.000	0.000	0.000	0.000	0.013	0.000
*Mg*μ4	129	0.000	0.000	0.000	0.010	0.000	0.000	0.000	0.000	0.000	0.000	0.000	0.000	0.000	0.000	0.000
	151	0.000	0.000	0.000	0.000	0.000	0.000	0.000	0.000	0.010	0.000	0.000	0.000	0.000	0.000	0.000
	153	0.000	0.000	0.000	0.000	0.000	0.000	0.000	0.000	0.000	0.000	0.070	0.000	0.000	0.000	0.000
	155	0.000	0.000	0.050	0.000	0.000	0.000	0.000	0.080	0.000	0.000	0.000	0.000	0.000	0.000	0.000
	159	0.000	0.000	0.000	0.000	0.000	0.000	0.000	0.000	0.000	0.000	0.000	0.070	0.000	0.000	0.000
	161	0.000	0.000	0.000	0.000	0.000	0.000	0.000	0.000	0.000	0.000	0.000	0.000	0.030	0.000	0.000
	162	0.000	0.000	0.000	0.010	0.000	0.000	0.000	0.000	0.000	0.000	0.000	0.000	0.000	0.000	0.000
	163	0.000	0.010	0.020	0.010	0.000	0.000	0.000	0.010	0.000	0.000	0.000	0.000	0.000	0.000	0.000
	165	0.060	0.030	0.020	0.140	0.010	0.070	0.100	0.000	0.060	0.000	0.170	0.070	0.060	0.000	0.030
	167	0.200	0.100	0.150	0.090	0.070	0.500	0.080	0.280	0.350	0.050	0.170	0.100	0.100	0.010	0.130
	169	0.060	0.040	0.050	0.030	0.010	0.040	0.100	0.090	0.040	0.080	0.030	0.100	0.000	0.000	0.100
	171	0.060	0.060	0.050	0.110	0.030	0.070	0.050	0.010	0.040	0.020	0.030	0.030	0.000	0.040	0.000
	173	0.200	0.160	0.330	0.240	0.260	0.140	0.220	0.240	0.130	0.250	0.200	0.330	0.000	0.050	0.200
	175	0.080	0.130	0.050	0.050	0.280	0.000	0.070	0.010	0.050	0.020	0.030	0.070	0.060	0.060	0.150
	177	0.070	0.110	0.130	0.110	0.120	0.110	0.040	0.100	0.120	0.220	0.000	0.130	0.000	0.200	0.180
	179	0.020	0.080	0.030	0.030	0.010	0.000	0.040	0.000	0.000	0.020	0.070	0.030	0.000	0.210	0.100

Table S1, cont.

Locus	Allele	MgJd	MgNd	MgRi	MgSa	MgOr	MgCt	MgCo	MgNo	MgYo	MgDi	H-MB	H-RF	McVc	MchCa	MeKa
	181	0.060	0.070	0.000	0.030	0.030	0.040	0.230	0.050	0.100	0.170	0.070	0.030	0.000	0.050	0.030
	183	0.140	0.120	0.020	0.060	0.140	0.000	0.030	0.050	0.010	0.050	0.000	0.000	0.030	0.300	0.030
	185	0.000	0.040	0.030	0.050	0.010	0.000	0.010	0.000	0.010	0.020	0.100	0.000	0.710	0.050	0.050
	187	0.020	0.020	0.030	0.030	0.030	0.000	0.010	0.010	0.040	0.050	0.000	0.000	0.000	0.010	0.000
	189	0.010	0.020	0.000	0.000	0.000	0.040	0.030	0.050	0.040	0.050	0.000	0.030	0.000	0.010	0.030
	191	0.000	0.000	0.000	0.010	0.000	0.000	0.000	0.000	0.000	0.000	0.000	0.000	0.020	0.000	0.000
	195	0.000	0.000	0.020	0.000	0.000	0.000	0.000	0.000	0.000	0.000	0.000	0.000	0.000	0.000	0.000
	197	0.000	0.000	0.000	0.000	0.000	0.000	0.000	0.030	0.000	0.020	0.070	0.000	0.000	0.000	0.000
	199	0.000	0.000	0.020	0.000	0.000	0.000	0.000	0.000	0.000	0.000	0.000	0.000	0.000	0.000	0.000
*Mg*µ5	116	0.000	0.011	0.000	0.000	0.000	0.000	0.000	0.000	0.000	0.000	0.000	0.000	0.000	0.013	0.000
	118	0.000	0.000	0.000	0.025	0.014	0.000	0.000	0.000	0.000	0.000	0.000	0.000	0.015	0.000	0.000
	120	0.000	0.045	0.000	0.000	0.000	0.000	0.000	0.000	0.000	0.000	0.000	0.000	0.000	0.000	0.000
	122	0.089	0.091	0.017	0.013	0.014	0.000	0.000	0.000	0.000	0.018	0.000	0.000	0.074	0.050	0.025
	124	0.089	0.148	0.100	0.013	0.108	0.000	0.000	0.000	0.000	0.000	0.000	0.000	0.162	0.063	0.150
	126	0.044	0.114	0.083	0.000	0.027	0.000	0.041	0.000	0.000	0.000	0.000	0.000	0.044	0.138	0.100
	128	0.011	0.193	0.050	0.000	0.014	0.000	0.000	0.000	0.000	0.018	0.000	0.000	0.000	0.038	0.000
	130	0.078	0.023	0.117	0.175	0.027	0.036	0.000	0.013	0.013	0.607	0.067	0.200	0.059	0.038	0.225
	132	0.600	0.045	0.383	0.450	0.703	0.143	0.284	0.538	0.688	0.250	0.667	0.533	0.618	0.413	0.400
	134	0.044	0.091	0.133	0.163	0.041	0.679	0.500	0.275	0.125	0.000	0.000	0.000	0.029	0.188	0.050
	136	0.011	0.114	0.033	0.025	0.014	0.036	0.054	0.038	0.025	0.054	0.067	0.100	0.000	0.038	0.050
	138	0.011	0.023	0.033	0.025	0.000	0.036	0.027	0.088	0.050	0.018	0.033	0.033	0.000	0.000	0.000
	140	0.022	0.057	0.033	0.088	0.041	0.036	0.014	0.025	0.025	0.036	0.133	0.033	0.000	0.025	0.000
	142	0.000	0.023	0.017	0.013	0.000	0.000	0.081	0.013	0.025	0.000	0.033	0.100	0.000	0.000	0.000
	144	0.000	0.000	0.000	0.013	0.000	0.000	0.000	0.013	0.050	0.000	0.000	0.000	0.000	0.000	0.000
	146	0.000	0.023	0.000	0.000	0.000	0.036	0.000	0.000	0.000	0.000	0.000	0.000	0.000	0.000	0.000
*Mg*µ8	184	0.000	0.000	0.000	0.000	0.000	0.000	0.000	0.000	0.000	0.000	0.000	0.000	0.000	0.000	0.025
	186	0.000	0.000	0.000	0.013	0.000	0.192	0.000	0.000	0.000	0.000	0.033	0.000	0.016	0.000	0.000
	188	0.000	0.023	0.000	0.013	0.000	0.077	0.000	0.000	0.000	0.000	0.000	0.000	0.000	0.050	0.000
	190	0.409	0.128	0.017	0.000	0.042	0.000	0.041	0.038	0.000	0.069	0.067	0.000	0.141	0.063	0.200
	192	0.125	0.023	0.083	0.013	0.125	0.077	0.041	0.038	0.026	0.155	0.000	0.091	0.188	0.175	0.175
	194	0.068	0.116	0.200	0.088	0.069	0.231	0.068	0.313	0.359	0.034	0.133	0.091	0.016	0.050	0.000
	196	0.080	0.105	0.083	0.013	0.125	0.115	0.135	0.025	0.026	0.103	0.200	0.182	0.047	0.063	0.075
	198	0.261	0.070	0.100	0.113	0.153	0.038	0.054	0.025	0.038	0.034	0.067	0.045	0.031	0.125	0.275
	200	0.034	0.209	0.050	0.088	0.111	0.000	0.068	0.050	0.051	0.103	0.000	0.045	0.203	0.363	0.175
	202	0.011	0.070	0.100	0.050	0.083	0.077	0.081	0.088	0.077	0.155	0.033	0.136	0.031	0.063	0.075
	204	0.000	0.093	0.100	0.100	0.042	0.077	0.014	0.000	0.051	0.121	0.000	0.045	0.031	0.000	0.000
	206	0.011	0.070	0.083	0.025	0.056	0.077	0.122	0.025	0.051	0.069	0.000	0.091	0.000	0.050	0.000
	208	0.000	0.035	0.033	0.163	0.042	0.000	0.122	0.113	0.077	0.103	0.067	0.045	0.000	0.000	0.000
	210	0.000	0.023	0.033	0.113	0.028	0.000	0.068	0.175	0.167	0.017	0.033	0.045	0.016	0.000	0.000
	212	0.000	0.000	0.033	0.075	0.056	0.038	0.068	0.013	0.026	0.000	0.067	0.000	0.000	0.000	0.000
	214	0.000	0.035	0.067	0.088	0.028	0.000	0.081	0.025	0.026	0.034	0.200	0.136	0.000	0.000	0.000
	216	0.000	0.000	0.000	0.050	0.042	0.000	0.027	0.075	0.013	0.000	0.000	0.045	0.281	0.000	0.000
	218	0.000	0.000	0.000	0.000	0.000	0.000	0.014	0.000	0.000	0.000	0.100	0.000	0.000	0.000	0.000
	220	0.000	0.000	0.017	0.000	0.000	0.000	0.000	0.000	0.013	0.000	0.000	0.000	0.000	0.000	0.000

Table S1, cont.

Locus	Allele	MgJd	MgNd	MgRi	MgSa	MgOr	MgCt	MgCo	MgNo	MgYo	MgDi	H-MB	H-RF	McVc	MchCa	MeKa
Mech8	173	0.000	0.000	0.000	0.000	0.000	0.000	0.000	0.000	0.000	0.000	0.033	0.000	0.000	0.000	0.000
	183	0.000	0.000	0.000	0.063	0.000	0.036	0.000	0.000	0.000	0.033	0.000	0.000	0.000	0.000	0.000
	185	0.000	0.000	0.000	0.000	0.000	0.000	0.000	0.000	0.000	0.000	0.000	0.000	0.000	0.138	0.100
	193	0.000	0.022	0.000	0.013	0.000	0.000	0.000	0.000	0.000	0.000	0.033	0.000	0.000	0.000	0.025
	195	0.000	0.000	0.000	0.050	0.000	0.000	0.014	0.025	0.025	0.050	0.067	0.033	0.029	0.013	0.000
	197	0.067	0.000	0.000	0.013	0.000	0.000	0.122	0.000	0.013	0.017	0.033	0.000	0.000	0.000	0.000
	199	0.000	0.000	0.033	0.100	0.000	0.036	0.230	0.000	0.038	0.217	0.333	0.200	0.500	0.075	0.025
	201	0.167	0.056	0.100	0.238	0.041	0.107	0.176	0.925	0.788	0.283	0.200	0.433	0.397	0.175	0.025
	203	0.433	0.567	0.467	0.363	0.689	0.536	0.297	0.013	0.125	0.317	0.200	0.267	0.074	0.463	0.525
	205	0.211	0.278	0.300	0.113	0.189	0.179	0.054	0.013	0.013	0.050	0.000	0.033	0.000	0.038	0.300
	207	0.100	0.067	0.033	0.038	0.054	0.107	0.068	0.013	0.000	0.017	0.033	0.033	0.000	0.025	0.000
	209	0.011	0.011	0.050	0.000	0.000	0.000	0.000	0.013	0.000	0.000	0.033	0.000	0.000	0.000	0.000
	211	0.000	0.000	0.017	0.000	0.027	0.000	0.041	0.000	0.000	0.000	0.033	0.000	0.000	0.075	0.000
	213	0.011	0.000	0.000	0.000	0.000	0.000	0.000	0.000	0.000	0.000	0.000	0.000	0.000	0.000	0.000
	215	0.000	0.000	0.000	0.013	0.000	0.000	0.000	0.000	0.000	0.017	0.000	0.000	0.000	0.000	0.000

Table S2. Genetic diversity of seven microsatellites in twelve samples of *M. galloprovincialis*, three samples of congeneric species (*M. trossulus* (MtVc), *M. chilensis* (MchCA) and *M. edulis* (MeKa)) and 2 samples from a *M. galloprovincialis – M. trossulus* hybrid zone (HgtMb and HgtRf).

**Sample**	**MgJd**	**MgNd**	**MgRi**	**MgSa**	**MgOr**	**MgCt**	**MgCo**	**MgNo**	**MgYo**	**MgDi**	**HgtMb**	**HgtRf**	**MtVc**	**MchCa**	**MeKa**
**N**	**45**	**45**	**30**	**40**	**37**	**14**	**37**	**40**	**40**	**30**	**15**	**15**	**34**	**40**	**20**
***Mg*µ1(21)**															
A (n)	16 (42)	18 (45)	10 (30)	9 (39)	13 (37)	5 (14)	8 (36)	9 (29)	9 (29)	1 1(30)	9 (14)	9 (15)	1 (34)	17 (40)	1 (20)
A private	1	1	-	-	1	-	-	-	-	1	1	-	-	-	-
A mode size	138	138	144	144	142	144	126	144	144	144	132	128	146	146	146
A size range	120-170	132-174	132-156	134-154	132-172	134-146	126-142	126-148	126-148	126-146	124-146	126-144	146	126-174	146
A null allele	0.202	0.108	0.193	0.016	0.141	-0.004	0.090	0.267	0.222	0.272	0.209	0.221	-	0.124	-
H_E	0.866	0.882	0.754	0.493	0.872	0.659	0.660	0.86	0.796	0.857	0.852	0.840	0	0.928	0
F_{IS}	0.450*	0.244*	0.470*	0.064	0.318*	0.025	0.242	0.599*	0.523*	0.611*	0.497*	0.524*	NA	0.273*	NA
***Mg*µ2(63)**															
A (n)	19 (45)	24 (45)	16 (30)	16 (40)	6 (37)	8 (14)	9 (37)	12 (38)	11 (40)	10 (30)	10 (14)	7 (12)	1 (34)	35 (39)	14 (14)
A private	-	1	-	-	-	-	1	-	-	1	-	-	-	18	-
A mode size	167	165	167	165	167	167	167	165	165	167	167	167	205	189-195	227
A size range	155-217	161-229	163-223	165-219	165-177	165-187	135-183	155-187	159-189	155-197	161-191	159-171	205	181-295	179-263
A null allele	0.179	0.151	0.229	0.256	-0.027	0.019	0.171	0.185	0.168	0.222	0.181	0.325	-	0.151	0.211
H_E	0.882	0.873	0.883	0.867	0.293	0.854	0.842	0.760	0.756	0.733	0.876	0.788	0	0.980	0.953
F_{IS}	0.396*	0.338*	0.509*	0.567*	-0.108	0.080	0.390*	0.446*	0.404*	0.545*	0.429	0.788*	NA	0.320*	0.476*
***Mg*µ3(71)**															
A (n)	5 (45)	5 (45)	6 (30)	5 (40)	2 (37)	3 (14)	6 (36)	2 (38)	2 (37)	4 (30)	2 (15)	3 (15)	6 (34)	4 (40)	3 (20)
A private	-	-	-	-	-	-	1	-	-	-	-	-	-	1	-
A mode size	138	138	138	138	138	138	140	138	138	138	138	138	140	140	140
A size range	132-142	132-142	138-142	138-144	138-140	136-140	132-142	138-140	138-140	132-142	138-140	138-142	132-144	138-146	138-144
A null allele	0.027	0.070	-0.022	0.074	0.058	-0.035	-0.095	-0.014	0.050	0.128	0.105	0.186	0.371	0.017	0.142
H_E	0.629	0.538	0.641	0.576	0.238	0.536	0.630	0.380	0.401	0.582	0.514	0.588	0.800	0.405	0.536
F_{IS}	0.082	0.215	-0.04	0.219	0.319	-0.067	-0.234	-0.039	0.191	0.370	0.352	0.547	0.853*	0.074	0.440

Table 2. cont.

Sample<br>N	MgJd<br>45	MgNd<br>45	MgRi<br>30	MgSa<br>40	MgOr<br>37	MgCt<br>14	MgCo<br>37	MgNo<br>40	MgYo<br>40	MgDi<br>30	HgtMb<br>15	HgtRf<br>15	MtVc<br>34	MchCa<br>40	MeKa<br>20
Mg**μ4(92)**															
A (n)	12 (42)	14 (45)	15 (30)	16 (40)	12 (37)	8 (14)	113 (37)	13 (40)	13 (39)	13 (30)	11 (15)	11 (15)	7 (34)	11 (40)	11 (20)
A private	-	-	2	-	-	-	-	-	1	-	1	1	1	-	-
A mode size	167173	173	173	173	175	167	181	167	167	173	173	173	185	183	173
A size range	165-189	163-189	155-199	129-191	165-187	165-189	165-189	155-197	151-189	167-197	153-197	159-189	161-191	167-189	165-189
A null allele	0.159	0.135	0.145	0.148	0.045	0.035	0.100	0.169	0.115	0.150	0.110	0.090	0.146	0.034	0.089
H_E	0.884	0.914	0.853	0.895	0.825	0.734	0.878	0.851	0.840	0.864	0.912	0.869	0.493	0.823	0.893
F_{IS}	0.354*	0.295*	0.336*	0.329*	0.116	0.124	0.230	0.383*	0.268*	0.344*	0.269	0.233	0.463*	0.088	0.216
Mg**μ5(331)**															
A (n)	10 (45)	14 (44)	11 (30)	11 (40)	10 (37)	7 (14)	7 (37)	8 (40)	8 (40)	7 (28)	6 (15)	6 (15)	7 (34)	10 (40)	7 (20)
A private	-	1	-	-	-	-	-	-	-	-	-	-	-	-	-
A mode size	132	128	132	132	132	134	134	132	132	130	132	132	132	132	132
A size range	122-140	116-146	122-142	118-144	118-140	130-146	126-142	130-144	130-144	122-140	130-142	130-142	118-134	116-140	122-136
A null allele	0.063	0.145	0.036	0.162	0.001	0.102	0.184	0.138	-0.030	0.040	-0.004	0.112	-0.004	0.192	0.057
H_E	0.621	0.902	0.813	0.743	0.496	0.538	0.671	0.636	0.511	0.575	0.545	0.683	0.589	0.779	0.774
F_{IS}	0.178	0.320*	0.098	0.395*	0.019	0.337	0.476*	0.371	-0.077	0.131	0.022	0.317	0.002	0.454	0.160
Mg**μ8(D5)**															
A (n)	8 (44)	13 (43)	14 (30)	15 (40)	14 (36)	10 (13)	15 (37)	13 (40)	14 (39)	12 (29)	11 (15)	12(11)	11 (32)	9 (40)	7 (20)
A private	-	-	-	-	-	-	-	-	-	-	-	-	-	-	1
A mode size	190	200	194	208	198	194	196	194	194	192202	196214	196	216	200	198
A size range	190-206	188-214	190-220	186-216	190-216	186-212	190-218	190-216	192.22	190-214	186-218	192-216	186-216	188-206	184-202
A null allele	0.096	0.125	0.069	0.106	0.009	0.133	0.053	0.047	0.013	0.070	0.073	-0.008	0.089	0.195	0.116
H_E	0.747	0.901	0.916	0.918	0.920	0.910	0.928	0.850	0.830	0.910	0.907	0.936	0.835	0.818	0.838
F_{IS}	0.239	0.278*	0.163	0.237*	0.034	0.324	0.126	0.117	0.042	0.166	0.192	0.029	0.214	0.450*	0.284

Table 2. cont.

Sample<br>N	MgJd<br>45	MgNd<br>45	MgRi<br>30	MgSa<br>40	MgOr<br>37	MgCt<br>14	MgCo<br>37	MgNo<br>40	MgYo<br>40	MgDi<br>30	HgtMb<br>15	HgtRf<br>15	MtVc<br>34	MchCa<br>40	MeKa<br>20
***Mech*8(D10)**															
A (n)	7 (44)	6 (43)	7 (30)	10 (40)	5 (36)	6 (13)	8 (37)	6 (40)	6 (40)	9 (29)	10 (15)	6 (11)	4 (32)	8 (40)	3 (20)
A private	1	-	-	-	-	-	-	-	-	-	1	-	-	-	-
A mode size	203	203	203	203	203	203	203	201	201	203	199	201	199	203	203
A size range	197-213	193-209	199-211	183-215	201-211	183-207	195-211	195-209	195-205	183-215	173-211	195-207	195-203	185-211	185-205
A null allele	0.124	0.135	0.085	0.087	0.052	0.094	0.086	0.059	0.100	0.037	0.072	0.136	0.202	0.129	0.044
H_E	0.736	0.603	0.691	0.793	0.492	0.687	0.817	0.146	0.368	0.780	0.831	0.731	0.600	0.735	0.641
F_{IS}	0.305*	0.373	0.229	0.212	0.176	0.272	0.206	0.486	0.389	0.102	0.198	0.362	0.559*	0.320	0.142

Note: N, sample size; *A*, number of alleles; n, number of individuals genotyped; *A* private, number of sample-specific alleles; *A* mode size, modal allele size (in base pairs); *A* size range, amplitude of the allelic series; *A* null allele, estimated frequency of null alleles; H_E, expected heterozygosity; F_{IS}, intrapopulation fixation index (Weir and Cockerham. 1984). P-value for F_{IS} was based on 10500 randomisations with an adjusted nominal level (0.05) for multiple comparisons, alpha = 0.00048.

Table S3. Pairwise estimates of genetic differentiation (D_{EST}, above diagonal) and gene distance (F_{ST}, below diagonal) between samples of *Mytilus* spp. All values except those bolded were significantly different from zero. The significance of D_{EST} was drawn from its 95% confidence interval (Suppl. Table 2). The significance threshold for F_{ST} was generated after 100 MC batches of 5,000 iterations each for alpha = 0.01.

Sample	MgJd	MgNd	MgRi	MgSa	MgOr	MgCt	MgCo	MgNo	MgYo	MgDi	HgtMb	HgtRf	MtVc	MchCa	MeKa
**MgJd**	-	0.220	0.222	0.281	0.292	0.335	0.353	0.371	0.352	0.329	0.246	0.296	0.584	0.389	0.279
**MgNd**	0.051	-	0.244	0.337	0.365	0.365	0.381	0.450	0.447	0.366	0.373	0.389	0.684	0.387	0.341
**MgRi**	0.047	0.041	-	0.077	0.223	0.145	0.301	0.229	0.228	0.177	0.247	0.160	0.645	0.451	0.322
**MgSa**	0.080	0.085	0.013	-	0.328	0.228	0.267	0.240	0.217	0.206	0.247	0.213	0.611	0.466	0.399
**MgOr**	0.098	0.131	0.095	0.138	-	0.409	0.439	0.420	0.424	0.275	0.332	0.218	0.682	0.470	0.386
**MgCt**	0.103	0.080	**0.040**	0.056	0.174	-	0.304	0.289	0.264	0.307	0.394	0.367	0.727	0.571	0.495
**MgCo**	0.094	0.090	0.082	0.098	0.179	0.092	-	0.365	0.385	0.296	0.215	0.225	0.645	0.466	0.507
**MgNo**	0.132	0.156	0.114	0.101	0.204	0.144	0.140	-	**0.013**	0.317	0.269	0.202	0.648	0.603	0.586
**MgYo**	0.113	0.146	0.095	0.078	0.186	0.133	0.138	**0.003**	-	0.319	0.259	0.250	0.637	0.611	0.582
**MgDi**	0.083	0.085	0.046	0.055	0.120	0.101	0.098	0.123	0.115	-	0.272	0.105	0.619	0.499	0.412
**HgtMb**	0.047	0.081	0.053	0.067	0.121	0.122	0.065	0.099	0.079	0.064	-	**0.066**	0.561	0.536	0.486
**HgtRf**	0.057	0.082	**0.042**	0.062	0.094	0.114	0.073	0.068	0.069	**0.025**	**0.003**	-	0.583	0.491	0.433
**MtVc**	0.268	0.284	0.296	0.298	0.398	0.361	0.304	0.347	0.337	0.305	0.288	0.310	-	0.549	0.471
**MchCa**	0.072	0.067	0.078	0.100	0.174	0.126	0.084	0.178	0.167	0.113	0.090	0.093	0.259	-	0.288
**MeKa**	0.095	0.092	0.112	0.153	0.223	0.163	0.173	0.252	0.238	0.146	0.155	0.158	0.249	0.094	-

Table S4. Confidence intervals (95%) for D_{EST} and F_{ST}.

Sample	MgJd	MgNd	MgRi	MgSa	MgOr	MgCt	MgCo	MgNo	MgYo	MgDi	HgtMb	HgtRf	MtVc	MchCa	MeKa
**MgJd**		0.170-0.281	0.167-0.280	0.235-0.330	0.234-0.353	0.260-0.407	0.280-0.419	0.314-0.434	0.294-0.409	0.258-0.402	0.162-0.342	0.223-0.383	0.530-0.633	0.324-0.445	0.222-0.341
**MgNd**	0.007-0.105		0.175-0.316	0.281-0.398	0.305-0.426	0.284-0.439	0.310-0.450	0.387-0.515	0.381-0.509	0.295-0.441	0.285-0.465	0.293-0.488	0.629-0.745	0.323-0.450	0.281-0.409
**MgRi**	0.000-0.096	0.007-0.080		0.015-0.137	0.155-0.298	0.058-0.231	0.225-0.370	0.169-0.298	0.160-0.306	0.106-0.256	0.160-0.332	0.082-0.278	0.585-0.706	0.393-0.516	0.264-0.386
**MgSa**	0.007-0.165	0.016-0.171	0.002-0.024		0.262-0.398	0.143-0.323	0.204-0.333	0.176-0.310	0.150-0.289	0.135-0.287	0.167-0.332	0.115-0.316	0.559-0.667	0.413-0.519	0.343-0.458
**MgOr**	0.041-0.168	0.033-0.243	0.038-0.171	0.056-0.229		0.320-0.499	0.368-0.510	0.362-0.481	0.354-0.492	0.199-0.350	0.233-0.435	0.131-0.338	0.635-0.730	0.412-0.522	0.331-0.445
**MgCt**	0.012-0.201	0.019-0.135	**(-0.002)-0.099**	0.01-0.113	0.078-0.285		0.222-0.389	0.196-0.389	0.184-0.349	0.223-0.388	0.285-0.491	0.259-0.488	0.647-0.805	0.497-0.642	0.407-0.572
**MgCo**	0.030-0.155	0.041-0.143	0.025-0.159	0.016-0.225	0.093-0.264	0.017-0.187		0.290-0.433	0.309-0.473	0.232-0.367	0.134-0.307	0.145-0.341	0.590-0.705	0.406-0.524	0.448-0.566
**MgNo**	0.036-0.254	0.041-0.322	0.019-0.276	0.026-0.203	0.061-0.403	0.031-0.320	0.056-0.241		**(-0.019)-0.067**	0.242-0.393	0.174-0.371	0.110-0.312	0.598-0.695	0.550-0.656	0.536-0.644
**MgYo**	0.038-0.191	0.043-0.264	0.029-0.209	0.031-0.133	0.061-0.330	0.018-0.267	0.067-0.206	**(-0.006)-0.015**		0.242-0.395	0.164-0.366	0.157-0.367	0.588-0.687	0.550-0.672	0.517-0.644
**MgDi**	0.035-0.145	0.032-0.142	0.012-0.089	0.016-0.097	0.048-0.214	0.014-0.217	0.019-0.191	0.034-0.234	0.045-0.207		0.171-0.367	0.018-0.214	0.553-0.685	0.426-0.570	0.338-0.487
**HgtMb**	0.011-0.083	0.022-0.144	0.011-0.094	0.006-0.162	0.044-0.210	0.024-0.230	0.012-0.133	0.029-0.230	0.029-0.153	0.016-0.139		**(-0.015)-0.191**	0.507-0.624	0.466-0.616	0.406-0.566
**HgtRf**	0.015-0.090	0.031-0.133	**(-0.005)-0.092**	0.000-0.163	0.039-0.163	0.026-0.210	0.019-0.137	0.027-0.147	0.043-0.094	**(-0.013)-0.072**	**(-0.008)-0.014**		0.529-0.648	0.405-0.579	0.359-0.515
**MtVc**	0.145-0.398	0.169-0.408	0.143-0.461	0.120-0.479	0.199-0.608	0.213-0.533	0.132-0.475	0.194-0.497	0.185-0.477	0.143-0.469	0.093-0.512	0.103-0.544		0.488-0.616	0.407-0.528
**MchCa**	0.046-0.098	0.042-0.099	0.038-0.109	0.044-0.170	0.063-0.310	0.083-0.166	0.059-0.112	0.083-0.296	0.109-0.234	0.068-0.469	0.071-0.112	0.063-0.131	0.133-0.379		0.221-0.368
**MeKa**	0.016-0.216	0.012-0.197	0.005-0.278	0.020-0.362	0.051-0.377	0.049-0.316	0.050-0.355	0.086-0.419	0.098-0.373	0.048-0.276	0.039-0.323	0.024-0.339	0.102-0.410	0.024-0.208	
